# Estimation of Tumor Interstitial Fluid Pressure (TIFP) Noninvasively

**DOI:** 10.1371/journal.pone.0140892

**Published:** 2016-07-28

**Authors:** Long Jian Liu, Stephen L. Brown, James R. Ewing, Brigitte D. Ala, Kenneth M. Schneider, Mordechay Schlesinger

**Affiliations:** 1 Department of Physics, University of Windsor, Windsor, Ontario, Canada; 2 Department of Radiation Oncology, Henry Ford Hospital, Detroit, Michigan, United States of America; 3 Department of Neurology, Henry Ford Hospital, Detroit, Michigan, United States of America; 4 Department of Radiology, Windsor Regional Hospital, Windsor, Ontario, Canada; 5 Department of Radiation Oncology, Windsor Regional Hospital, Windsor, Ontario, Canada; University of Arizona, UNITED STATES

## Abstract

Tumor interstitial fluid pressure (TIFP), is a physiological parameter with demonstrated predictive value for a tumor’s aggressiveness, drug delivery, as well as response to treatments such as radiotherapy and chemotherapy. Despite its utility, measurement of TIFP has been limited by the need for invasive procedures. In this work, the theoretical basis for approaching the absolute value of TIFP and the experimental method for noninvasively measuring TIFP are presented. Given specific boundary and continuity conditions, we convert theoretical variables into measurable variables by applying MRI technology. The work shows that TIFP in the central region of the tumor can be estimated by an analysis of the variation of tissue fluid motion in the tumor rim and surrounding tissue. It is determined from three noninvasive measurable parameters: i) an estimate of the velocity of the tumor interstitial fluid at the tumor surface, which is maximal, ii) a measurement of the distance from the tumor surface to where the tumor exudates are absorbed (or normalized), and iii) an estimate of the hydraulic conductivity of the interstitium through which the tumor exudate travels. We experimentally show that the fluid flow within the tumor rim is not uniform, even for a round shaped tumor, and demonstrate the procedures for the noninvasive measurement of TIFP.

## Introduction

Despite significant strides in the treatment of solid tumors in tissues such as breast and prostate, local control of other cancers such as primary brain tumors and pancreatic cancer remain challenging. The determinants of local tumor control are complex and multifactorial including microscopic spread beyond traditionally accepted clear margins at surgical resection, inherent radio-resistance and chemo-resistance in both the primary and ‘adjuvant’ (i.e postoperative) use of these therapies and a wide range of tumor and host biological characteristics that are factors in the growth and potential control of malignant tumors. As new therapies are developed and medicines become increasingly individualized, a need arises for an effective and early predictor of response. Tumor interstitial fluid pressure (TIFP) is a physiological parameter with demonstrated predictive value for a tumor’s aggressiveness, as well as response to chemotherapy and radiotherapy [1–7]. However, its clinical use has been limited because the technique available for its measurement has been invasive.

Because of its clinical importance, a noninvasive estimate of TIFP, if such could be developed, might have a profound effect on the assessment and treatment of many solid tumors. By using GdDTPA contrast enhanced MRI, Hassid et al. [8] found that an elevated TIFP of human lung tumors in the central region is associated with a decreased concentration of contrast agent. This shows that the elevated TIFP hinders drug delivery, and may be an indication that a noninvasive measurement of TIFP can be developed. However, to date there is no quantitative relation between TIFP and tissue contrast agent concentration. We will examine the flow of tumor exudate in and around its vascular sources and establish a theoretical framework for a noninvasive estimate of TIFP.

We had previously suggested a positive linear relation between TIFP in the central region and fluid velocity at the tumor surface [9]; an index of TIFP can be formed by noninvasively measuring the fluid velocity at the tumor surface. Supporting the importance of this measurement is a finding [7] that primary tumors in metastasis-positive mice displayed higher IFP and fluid velocity than those in metastasis-negative mice. Similarly, the same investigators found that the fluid velocity at the tumor periphery in cervical cancer patients with pelvic lymph node metastases was faster than fluid velocity in cancer patients without lymph node involvement. The fluid velocity at the tumor surface may be used as a biomarker of tumor aggressiveness [7]. It has been proposed [7, 9] that tumor exudate fluid flow velocity has a negative linear relation with the distance from the tumor surface in the region outside the tumor. Studies have shown that TIFP decreases steeply in the periphery [1, 10], and that fluid velocity increases from zero centrally to a maximum at the tumor surface, in accordance with Darcy’s law, which posits a relationship between fluid velocity in a porous medium and the local gradient of interstitial pressure.

The purpose of this paper is to present a practical model that may be applied for a quantitative noninvasive measurement of TIFP. Experiments (see below) show that the fluid flow within the tumor rim is not uniform, even for a round shaped tumor. A theoretical basis for a TIFP noninvasive measurement is formulated for locally one-dimensional geometries often encountered in practice. The variation of interstitial fluid flow in the tumor rim is described and visualized through MRI images. The relationship between TIFP, velocity, maximum exudate distance, and fluid conductivity is derived, and the noninvasive measurement of these variables with DCE-MRI is demonstrated. This work provides a theoretical basis for the very useful empirical relationship proposed by Hompland et al. [7] to relate the change with time in the position of the point of peak contrast outside a tumor.

## Materials and Methods

### Model description

We divide a tumor into three regions: 1) The central region, which contains a necrotic core. 2) Tumor periphery region. In this region, tumor blood vessels are plentiful. This region is the main source of tumor interstitial fluid. 3) The intermediary region [9].

We assume that there are no (or little) functional lymphatics or drainage vessels in the central region, but a functional drainage system exists at the periphery. If the drainage ability within the tumor (mainly from tumor periphery) is greater than or equal to fluid source (*J*_S_≤*J*_Lmax_), the net fluid flow at the tumor surface is zero. In this case, the interstitial fluid flow is limited to the tumor and TIFP at the surface (and outside it) is equal to the pressure of the surroundings, which is usually near atmospheric pressure in normal tissue. This case is defined as that of an “isolated” tumor [9, 10]. The following case also belongs to an isolated tumor: there are no functional drainage vessels such as lymphatics surrounding the tumor and no way for fluid to flow out. This condition may only exist in a malignant tumor and TIFP might be very high and could even reach the extreme value *p*_m_ [9], a maximum pressure barrier. An “embedded” tumor is defined as the case in which the tumor is enclosed by normal tissue and *J*_S_>*J*_Lmax_, *i*.*e*., fluid exits the tumor surface, *R*_s_, and flows into the normal tissue. Lymphatic vessels (and/or other drainage channels) are numerous and functional in normal tissue, forming a functional drainage system. One defines the maximum position *R*_m_ that tumor interstitial fluid can spread to as the place at which the pressure becomes the same as that of normal tissue.

The interstitial fluid pressure *p*_0_ within the central region is uniform in the steady state, as both we and others have described [1, 9, 10, 11], and by Darcy’s law the fluid velocity is zero. The boundary of the central region, *R*_0_, is an iso-pressure and iso-velocity surface. In the intermediary region, there is almost no tumor fluid source and only a drain for tumor fluid to be absorbed or normalized. This gives the boundary conditions at the interface, *R*_m_. TIFP is the same as the pressure *p*_∞_ in the normal tissue or surrounding environment; tumor interstitial fluid velocity is zero there.

**Fig 1:**
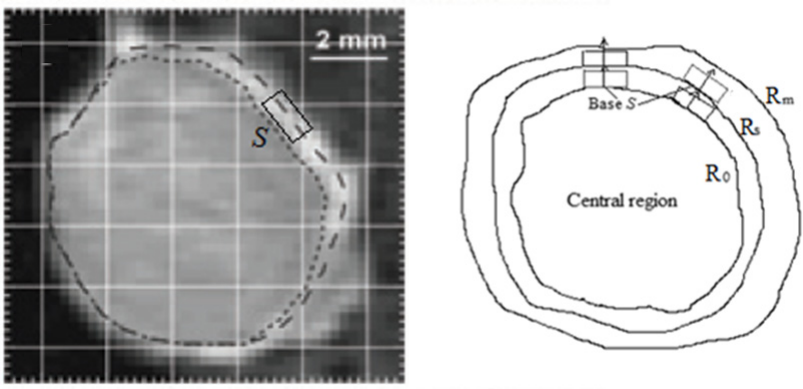
Schematic of fluid flow in the regions close to tumour surface. A tumour boundary can be chosen so that it is locally one-dimensional (the figure on the left is from Fig 2B-4 in Ref. 7; it shows a T1-weighted MRI image of a TS-415 cervical carcinoma). *S* represents the base of the fluid flux in the local coordinate system in which a 1D model can be applied.

Though we assign *R*_0_, *R*_s_ and *R*_m_ three parameters to represent the interfaces of various regions, the interfaces do not necessarily have to be spherical in shape as shown in Fig 1. In practicality, most tumors are irregular in shape and the curvature of a tumor varies locally. Also, tumor vascular vessels and their permeability are not uniform. Therefore, even a spherically shaped tumor may not give a spherically symmetrical distribution of TIFP. Usually, one surface area may have a large curvature, while another may have a small curvature that is close to zero. Similarly, the vasculature is distributed mostly in the periphery of the tumors [10] and is an irregular distribution of fluid sources. To take the fluid source networks generated by the arteriole-venule pairs as locally one-dimensional may be more practical and realistic, especially when the width of the vascularized peripheral region is relatively narrow compared to the overall dimensions of the tumor.

**Fig 2:**
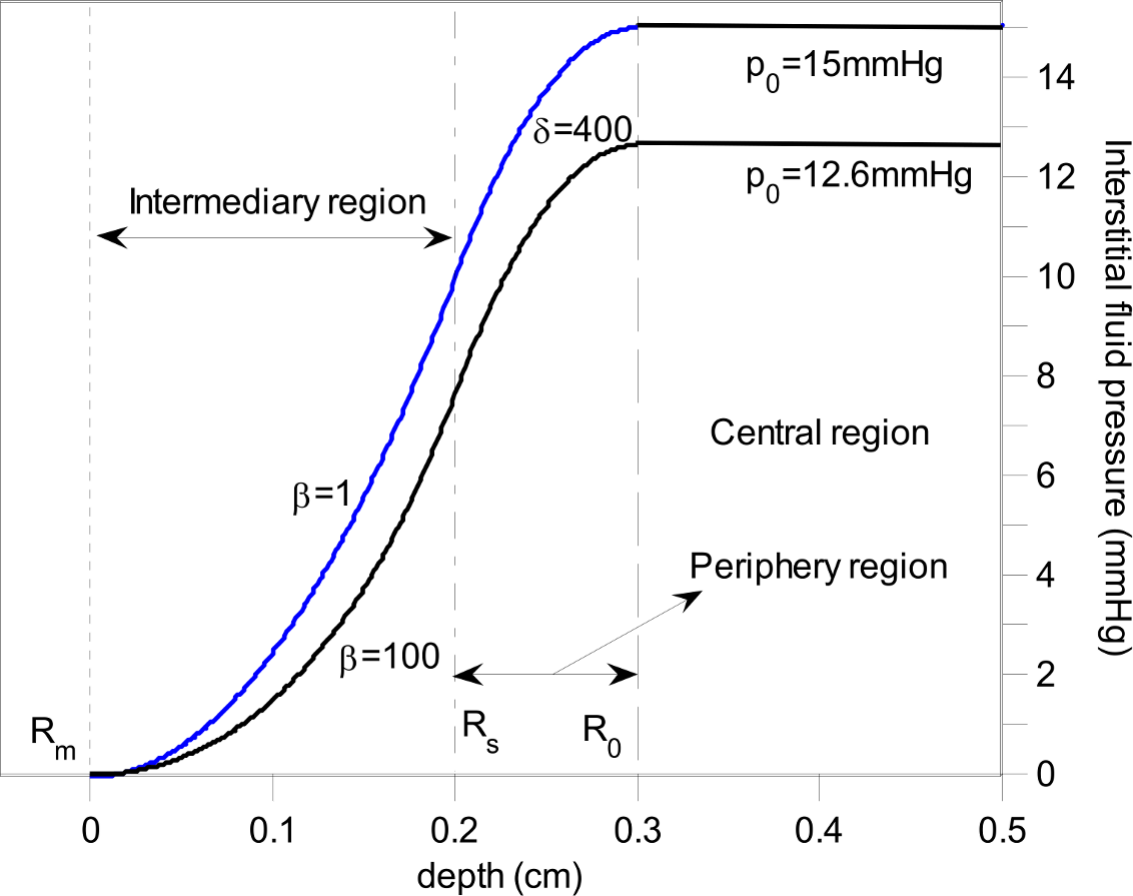
Comparison of TIFP distribution as a function of position (depth) at steady state for various *β*. Other parameters are the same based on Eq 18 in appendix.

We have derived the distributions of TIFP and fluid velocity in a one-dimensional case in the appendix. Fig 2 shows TIFP distribution for various vascular and lymphatic conditions. For a tumor with poor drainage system (e.g. malignant tumor), the solution for TIFP distribution is approximated as:

$$p(r)=\{\begin{array}{cc} p_{0}-\frac{u(R_{s})}{2K^{\prime }d_{0}}{\left(r-R_{0}\right)}^{2} & \left(R_{0}<r<R_{s}\right)\\ \frac{u(R_{s})}{2Kd_{m}}{\left(R_{m}-r\right)}^{2}+p_{\infty } & \left(R_{s}<r<R_{m}\right) \end{array}$$ (1)

Correspondingly, the fluid velocity is expressed as:

$$u(r)=\{\begin{array}{cc} \frac{u(R_{s})}{d_{0}}(r-R_{0}) & \left(R_{0}<r<R_{s}\right)\\ \frac{u(R_{s})}{d_{m}}(R_{m}-r) & \left(R_{s}<r<R_{m}\right) \end{array}$$ (2)

$$p(R_{s})=\frac{u(R_{s})d_{m}}{2K}+p_{\infty }\;\mathrm{and}\;p_{0}=\frac{u(R_{s})d_{0}}{2K^{\prime }}+p(R_{s})$$ (3)

where *d*_m_ = *R*_m_-*R*_s_, and *d*_0_ = *R*_s_-*R*_0_, *p*_∞_ is the pressure of the surrounding tissue, usually zero relative to the atmosphere, *K* and *K*′are the high hydraulic conductivities of interstitium in the normal tissue and tumor respectively. All the variables are measurable. In the case of an “isolated” tumor as we discussed above, no exudate crosses the tumor surface. The following conditions are satisfied: *p*(*R*_0_) = *p*_0_, d*p*(*R*_0_)/d*r* = 0 and *p*(*R*_s_) = *p*_∞_ (*d*_m_ = 0). Thus, we have

$$p(r)=p_{0}-\frac{p_{0}-p_{\infty }}{d_{0}^{2}}{\left(r-R_{0}\right)}^{2}\;\left(R_{0}<r<R_{s}\right),$$ (4)

$$u(r)=\frac{2K^{\prime }(p_{0}-p_{\infty })}{d_{0}^{2}}(r-R_{0})\;\left(R_{0}<r<R_{s}\right),$$ (5)

$$\mathrm{and}\;p_{0}=p_{\infty }+\frac{u(R_{s})}{2K^{\prime }}d_{0}.$$ (6)

Based on Eqs 3 and/or 6, once we know the hydraulic conductivity, the fluid velocity and the size of the tumor rim, we can estimate TIFP.

A contrast agent is commonly used to study interstitial fluid flow. As a tracer, it reflects (visualizes) interstitial fluid flow. We assume that the contrast agent does not affect interstitial fluid flow and instead becomes a part of the fluid. Interstitial fluid flow is relatively steady for a tumor at a specific stage (e.g. the fluid velocity at a specific point today is almost the same as that of tomorrow). However, when the interstitial fluid with contrast agent flows from tumor surface *R*_s_ to an arbitrary position *r* through time *t* continuously, the fluid velocity becomes the same at that position, *u*(*t*) = *u*(*r*). For the sake of convenience, we let *s* = *r*(*t*)−*R*_*s*_ which is defined as the distance that the contrast agent travels from the tumor surface. Therefore, we have (for the tracer):

$$u(s)=u(t)=ds/dt=u(R_{s})(1-s/d_{m}),$$ (7)

$$s(t)=d_{m}[1-e^{-u(R_{s})t/d_{m}}].$$ (8)

The circle that is composed of the brightest points in a specific direction within the tumor rim at various times can be determined. Therefore, both the *u*(*R*_s_) and *d*_m_ can be estimated through curve fitting. The derivative of Eq 8 is

$$u(t)=u(R_{s})e^{-u(R_{s}t/d_{m})}.$$ (9)

It shows that the fluid velocity decreases exponentially. When *t* = 0, *r*(*t*) = *R*_s_, *s*(*t*) = 0, *u*(*t*) = *u*(*R*_s_); *t*→∞, *r*(*t*) = *R*_m_, *s*(*t*) = *d*_m_, *u*(*t*) = 0. It is consistent with the description above.

### Noninvasive measurement of tumor interstitial fluid velocity based on DCE-MRI images

Eq 9 indicates a method for measuring the interstitial fluid velocity at tumor surface *u*(*R*_s_). Tumor surface can be determined by find the circle which is composed of the brightest points in various directions before contrast agent is applied or within a short time after contrast agent is applied. We can then measure the displacement of circle after a certain time *t* delay. Through curve fitting based on Eq 8, we can find *u*(*R*_s_).

**Fig 3:**
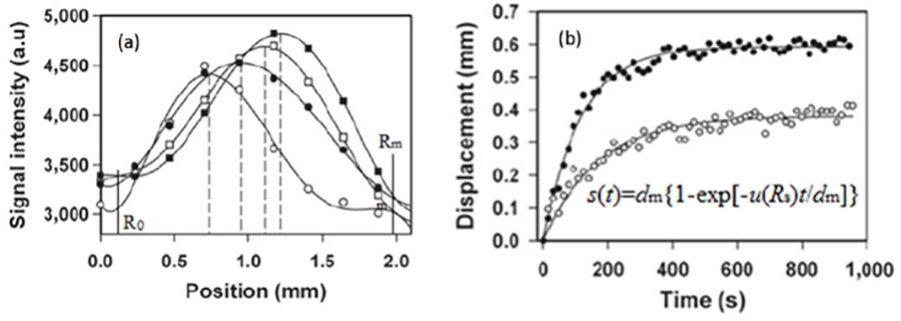
Method to determine *d*_m_ and *u*(*R*_s_). (a) Signal intensity vs. position in a tumor periphery at 15, 80, 110 and 450 seconds respectively. (b) Curve fitting for determining *d*_m_ and *u*(*R*_s_) for a tumor with low IFP (lower) and a tumor with high IFP (upper) [7].

For a specific tumor, the data can be measured from a chosen direction. After the bolus administration of a contrast agent, an estimate of the peak position *versus* time can be fitted to estimate the value of *d*_m_ and *u*(*R*_s_), as shown in Fig 3. Also, the position of *R*_0_ can be aligned based on the signal intensity vs. position, as shown in Fig 3(A). Fluid within *R*_0_ is still and DCE-MRI has the least intensity of pixels. Similarly, we may be able to locate the position of *R*_m_. *R*_s_ is determined by find the brightest point before contrast agent is applied or within a short time, which is long enough for visualizing the tumor rim, after contrast agent is applied. In fact, *R*_s_ may also be determined by curve fitting.

### Measurement of TIFV based on phase contrast imaging

Phase contrast imaging is a tool for visualizing fluid flow. Spins that are moving along the direction of magnetic field gradient causes a phase shift proportional to their velocity: *ϕ* = *γMu* + *ϕ*_0_ = *γG*(*T*/2)^2^*u* + *ϕ*_0_, where γ is the gyromagnetic ratio, M the momentum, G represents the magnetic gradient, T the bipolar gradient duration, and *ϕ*_0_ is the background phase effect due to susceptibility of field inhomogeneity [12]. It requires two repetitions of the sequence, the second of which used bipolar gradients of opposite polarity to the first. Measuring two data sets, we can have the velocity: *u* = Δ*ϕ*/(*γ*Δ*M*). By comparing the phase of signals from each location in the two sequences the exact amount of motion induced phase change can be determined to have a map where pixel brightness is proportional to spatial velocity. Regions that are stationary remain black while moving regions are represented as gray to white.

However, the method based on a velocity contrast sequence may assume that the concentration of the contrast agent is spatially and temporally uniform. Actually, the agent concentration may change with time and the gray value is correlated with the agent concentration. Note that even though the gray value is proportional to the fluid velocity, the proportional coefficient may relate to the concentration of the contrast agent. Therefore, even though the fluid velocity is the same, the gray value may vary if the concentration of the contrast agent is different. Usually, the concentration of contrast agent is time dependent. Therefore, the gray value is also time dependent as shown in Tables 1–3. However, in the same slice with a certain time delay, the concentration of the contrast agent is fixed. Thus, the velocity of the interstitial fluid is proportional to the average gray value in a specific direction within the tumor rim. Through post-processing, phase contrast imaging can give maps of fluid velocity streamlines [13]. As we know that the interstitial fluid velocity at the tumor surface is maximal. This way we can find the fluid velocity at tumor surface *u*(*R*_s_) in a specific direction. Measuring fluid velocity noninvasively is an important step for actualizing TIFP noninvasive measurement.

**Table 1:** The average gray-value of the rim of tumor 1 in liver 1 in different directions at various times.

	North	NW	West	SW	South	SE	East	NE
**A5: 0 s**	134.2	122.2	114.3	122.3	122.2	139.2	136.2	138.6
**A7:60s**	120.4	109	104.8	102.3	115.2	105.4	107.4	100.9
**A8:90s**	128.6	120.1	123	127.8	147.2	137.1	147.6	142.2
**A9:120s**	133.2	133.4	130	130	139.1	134.3	150.4	148.6
**A10:150s**	143.3	139.5	138.5	135.6	147.7	147	143.2	144.4
**A13:5min.**	148.3	144.4	141.5	147.6	162.1	154.2	162.2	155.7
**A14:10min.**	173.3	159	152.3	151	170.2	164	185.7	178.8

**Table 2:** The average gray-value of the rim of tumor 2 in liver 1 in different directions at various times.

	North	NW	West	SW	South	SE	East	NE
**A5: 0 s**	140	141.2	144.2	147.6	156.4	158	150.7	152.7
**A6:30 s**	147.3	148.2	140.3	138.7	140.3	141.6	144.5	151.2
**A7:60 s**	131.1	128	116	113	112.3	114	110	118
**A8:90 s**	149.7	150	147.7	153.2	149.9	149.6	146.3	149
**A9:120s**	157.9	152.2	151.8	151.3	149.3	149.4	146.8	148.4
**A10:150s**	161.2	161.6	156.1	147.5	143	150.4	146.2	147.6
**A13:5min.**	164.5	163.8	166.7	163.1	156.2	154.7	160.9	161.6
**A14:10min.**	174.8	175.9	179.3	176.2	178.2	179.1	178.5	180

**Table 3:** The average gray-value of the rim of tumor 1 in liver 2 in different directions at various times.

	North	NW	West	SW	South	SE	East	NE
**A6: 60s**	206.8	200.8	203.3	204	203.9	205.4	212.9	205.4
**A7: 90 s**	231	215.5	219	215.1	210	213.3	225.5	224.6
**A8: 120 s**	234.4	220	221.4	219.4	223.1	220.2	224.6	226.4
**A9: 150 s**	211.1	211.8	199.6	204	185.4	196.4	209.3	207.6

## Results

**Fig 4:**
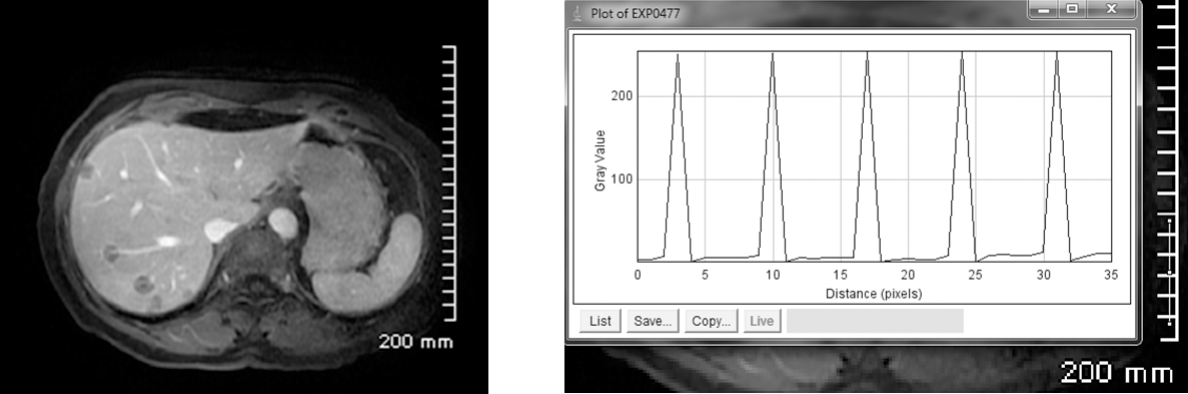
Schematic for determining the unit length in mm per pixel.

**Fig 5:**
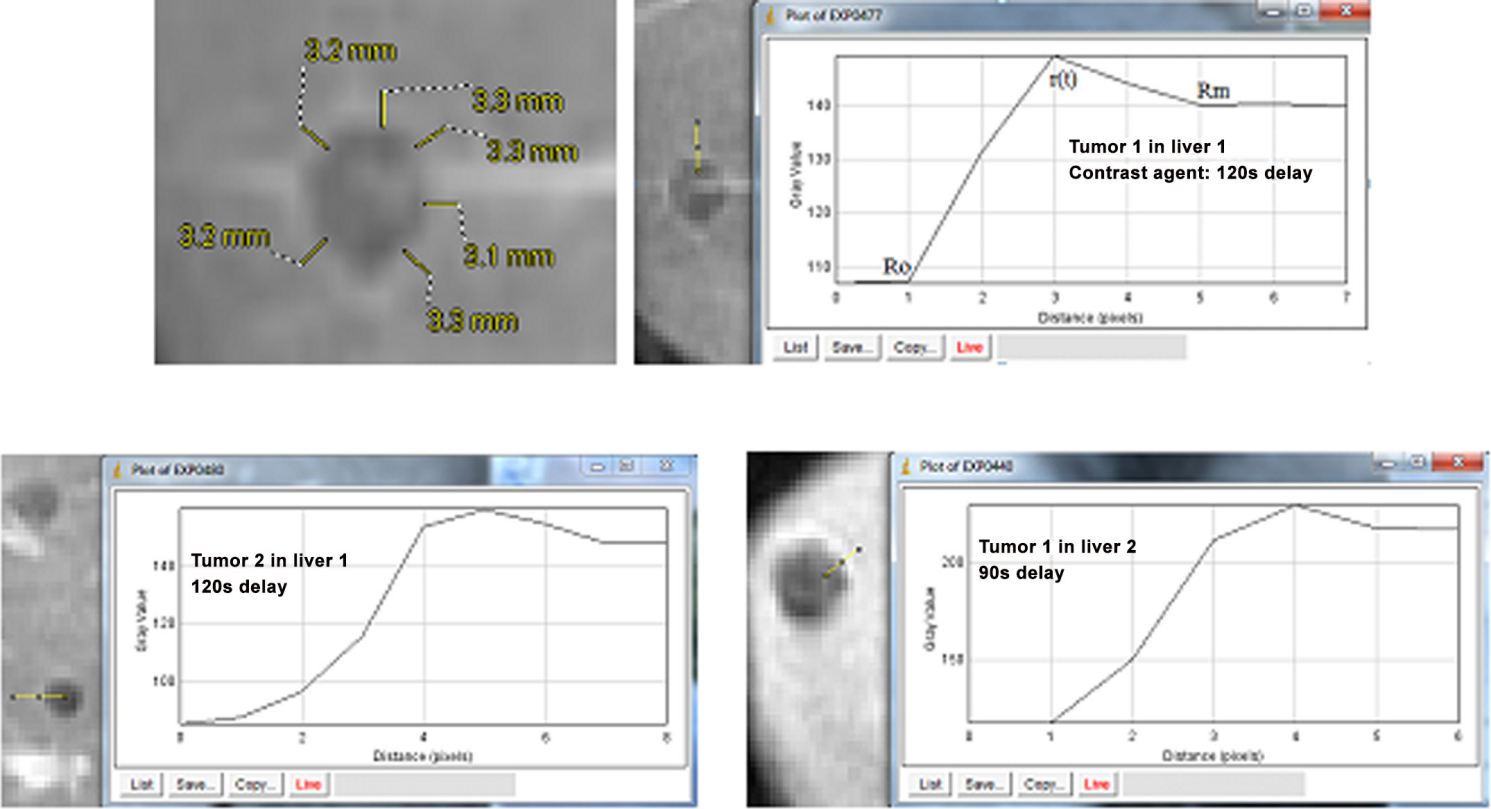
Liver tumors with a contrast enhancing rim visible. Several measurements of the rim were made along tumor periphery to infer TIFP. A judicious local coordinate system may be chosen, in which a 1D model can be applied. The rim of tumor 1 in liver 1 is approximately 3.2 mm in 4 pixels, ~0.8mm/pixel.

DCE-MRI images of tumors in human livers were obtained for study and analysis. We can covert the pixels to millimeters. The length in mm per pixel is determined by measuring the known length of scale in pixel as shown in Fig 4. The contrast agent visualizes the rim variation at tumor periphery. Three tumors (tumors 1 and 2 in liver 1, and tumor 1 in liver 2) are measured as shown in Fig 5. The tumor rim (*R*_s_-*R*_0_) or *R*_m_-*R*_0_ can be measured although the accuracy may be improved by using a proper method and specifying and standardizing the process. The position of *R*_0_ can be aligned based on the signal intensity vs. position. Fluid within *R*_0_ is still and DCE-MRI has the least intensity of pixels. Similarly, we can locate the position of *R*_m_, where the intensity of the signal is the least again. The gray value—distance (pixel) distribution is measured by using the software “ImageJ”. Although the images are too coarse (~0.8mm/pixel) for measuring the *r*(*t*) accurately, Fig 5 clearly shows that this method is practical for determining the positions of *R*_0_, *r*(*t*) and *R*_m_, which are vital parameters for TIFP noninvasive measurement.

We examined the time delay MRI images without and with contrast agent in 0s (without contrast agent), 30s, 60s, 90s, 120s, 150s, 5mins, and 10 minutes for tumors 1 and 2 in the same host liver but different slices. Tables 1 and 2 show the average gray value within the tumor rim at different times for tumors 1 and 2 respectively. For tumor 1, the images with 30s delay do not show a clear tumor rim. The gray-value variation cannot be measured, and it is ignored in Table 1.

The MRI images clearly show that the contrast between the rim and the background decreases as time increases from 150s to 10min. The change rate of concentration of contrast agent should be correlated with the fluid velocity. Therefore, the concentration of contrast agent at different time may predict the fluid velocity.

We also measured the average gray value along the rim in specific directions of tumor 1 in liver 2. The results are listed in Table 3.

However, the data in Tables 1 and 2 show that the gray values decrease in the first 60s after contrast agent is applied. The gray value then increases, though some fluctuation occurs for data in Table 1. The change in gray value may indicate the variation of the contrast agent concentration.

For tumor 1 in liver 2, contrast starts from A5. However, the rims could not be seen from A1 to A5 (until the images with 60s delay after contrast agent is applied). Also, the rims cannot be seen with 5 and 10 min delay. From Table 3, we can see that the gray value of the tumor rim increases from 60 s to 120 s, and then it decreases at 150 s.

The experiment data show that the fluid flow within the tumor rim is not uniform, even though the shape of the tumor is fairly round. This is because the leakage and permeability of tumor blood vessels are not uniform. Therefore, a spherical model usually cannot be applied to predict the interstitial fluid velocity distribution and the pressure distribution. In this case, as we have presented, the one-dimensional model under a judicious local coordinate system can be applied for approaching the tumor interstitial fluid pressure in the central region.

TIFP correlates with hydraulic conductivity. Boucher et al. [14] developed a method for estimating the hydraulic conductivity of tumor interstitium in vivo by measuring the radial pressure difference based on a spherical model. However, as we showed above, the conditions for a spherical model are usually not satisfied, even for a spherically shaped tumor. Hompland et al. [7] experimentally showed the linear relation between fluid velocity *u*(*R*_s_) and TIFP based on linear regression analysis of their experimental data. The slope *k* can be found for various tumors and contrast agents as shown in Table 4.

**Table 4:** The slope of *u*(*R*_s_)~*p*_0_ for various tumors and contrast agents.

Contrast agent	Gd-DTPA			Gadomelitol		
**Tumor**	TS-415	U-25	Together	TS-415	U-25	Together
***k*(mm/mmHgs)**	1.29x10^-4^	1.31 x10^-4^	1.30 x10^-4^	1.54 x10^-4^	1.45 x10^-4^	1.52 x10^-4^

The slope is described as $k=\frac{2K^{\prime }}{d_{0}}$ for an isolated tumor (*d*_m_ = 0) and $k=\frac{1}{\frac{d_{m}}{2K}+\frac{d_{0}}{2K^{\prime }}}$ for an embedded tumor ($k=\frac{2K^{\prime }}{R_{m}-R_{0}}$, if the hydraulic conductivities in the periphery region from *R*_0_ to *R*_s_ within the tumor and the intermediary region from *R*_s_ to *R*_m_ are approximately the same *K* = *K*′). The result shows that the ratio of the hydraulic conductivity to the rim size is nearly the same for different tumors. It is related to the types of contrast agents. For a tumor with a 2 mm rim, the hydraulic conductivity of tumor interstitium is around 1.30x10^-6^ cm^2^/mmHgs for Gd-DTPA and 1.50x10^-6^ cm^2^/mmHgs for Gadomelitol. We should choose a proper contrast agent that can best represent interstitial fluid flow. Contrast agent Gd-DTPA gives a better result.

Usually, the hydraulic conductivity in normal tissue is steady since the structure of normal tissue is stable. The hydraulic conductivity within a certain type of normal organ (tissue) should be almost always the same. However, the interstitium structure within the tumor may change with the progression of the tumor stage. Thus, the hydraulic conductivity within the tumor may depend on the tumor and its stage. Even so, based on our method, we can estimate *R*_m_-*R*_0_, *d*_m_ and *d*_0_. The fluid velocity can also be found. Usually, the hydraulic conductivity *K* and the fluid pressure *p*_∞_ are known in a specific normal organ tissue. For instance, IFPs of normal tissues are usually as follows: -8mmHg in the lungs, -3 or -2 mmHg in subcutaneous tissues, 0 to +2 mmHg in the liver and kidneys, and +6 mmHg in the brain [15]. Therefore, the hydraulic conductivity *K*′ and TIFP within the tumor can be estimated. Generally, a malignant tumor with a higher TIFP causes a larger *d*_m_. Also, a malignant tumor may have a larger hydraulic conductivity. A larger necrotic core may lead to a smaller *d*_0_. Therefore, the slope *k* should not change much. Once we find the fluid velocity *u*(*R*_s_) and the pressure in the host tissue, we can estimate TIFP.

For the data shown in Fig 3(B), we fit the two cases with the following two functions:

*s*_*high*_(*t*) = 0.60(1−*e*^−0.0053*t*/0.6^) for the high TIFP, and *s*_*low*_(*t*) = 0.39(1−*e*^−0.0023*t*/0.39^) for the low TIFP case. Based on the data in Fig 3(A), we estimate that *R*_m_-*R*_0_ = 1.86mm. If *d*_m_ = 0.60mm, then *d*_0_ = 1.26mm; if *d*_m_ = 0.39mm, then *d*_0_ = 1.47mm. The fluid velocity *u*(*R*_s_) is 5.3x10^-3^mm/s and TIFP is approximately 40.8 mmHg for the upper curve case; *u*(*R*_s_) is 2.3x10^-3^mm/s and TIFP 17.7 mmHg for the lower case in Fig 3(B). It may be possible to directly measure *d*_m_. We may approach the results more accurately by combining this with curve fitting.

**Fig 6:**
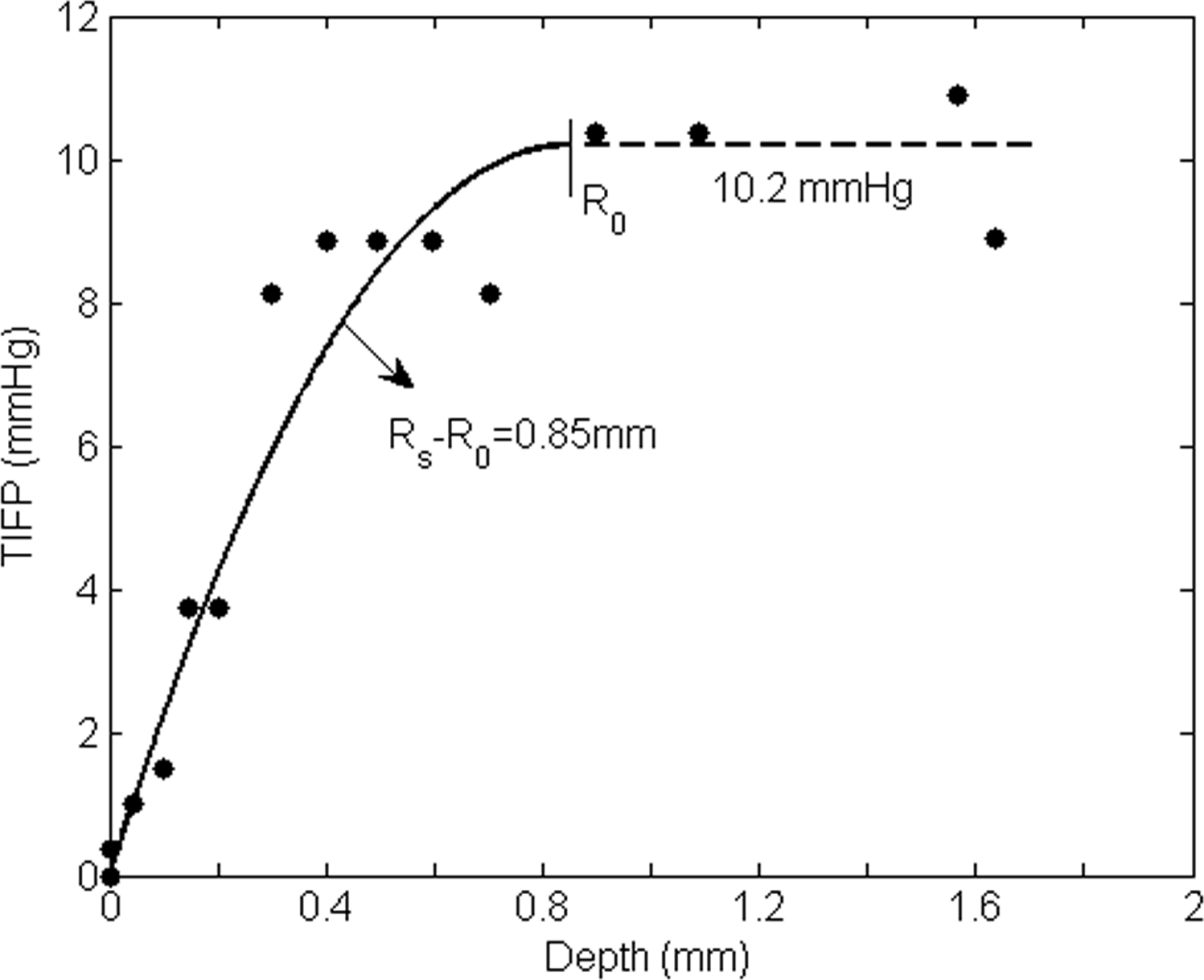
Comparison of the model for an ‘isolated’ tumor based on Eq 4 with the experimental data from Fig 8 in Ref. 1. Only three parameters are needed and they are measurable.

Boucher et al. [1] applied Baxter and Jain’s mathematical model [10] to fit one set of their experimental readings for subcutaneous mammary adenocarcinoma. Though there are only two explicit parameters (*α* and *P*_e_, see Eq 8A of Ref. 9), more parameters are needed for determining these two. Also, the tumor radius *R* (we use *R*_s_) must be known in order to do curve fitting. The two explicit parameters are theoretically given. They are difficult to determine in practical application. However, when Eq 4 is employed to fit the experimental points from Fig 8 of Boucher et al. [1], only three parameters are needed. Most importantly, these three parameters are measurable. This is essential for noninvasive measurement. Curve fit to the data in Fig 6 is obtained, with an estimate of the distance (*R*_s_-*R*_0_) of 0.85mm. In this case, we get *K*′ = 5.53×10^−7^ cm^2^/mmHgSec and *u*(*R*_s_)≈1.33μm/s.

Though many models take the hydraulic conductivities inside and outside a tumor as two different constants [10, 11, 16, 17], some take them to be the same [18, 19]. The hydraulic conductivity in the periphery region and that in the intermediary region should not differ from each other significantly. In this case, based on Eq 3, TIFP can be estimated from the following:

$$p_{0}=\frac{u(R_{s})(R_{m}-R_{0})}{2K^{\prime }}+p_{\infty }.$$ (10)

**Fig 7:**
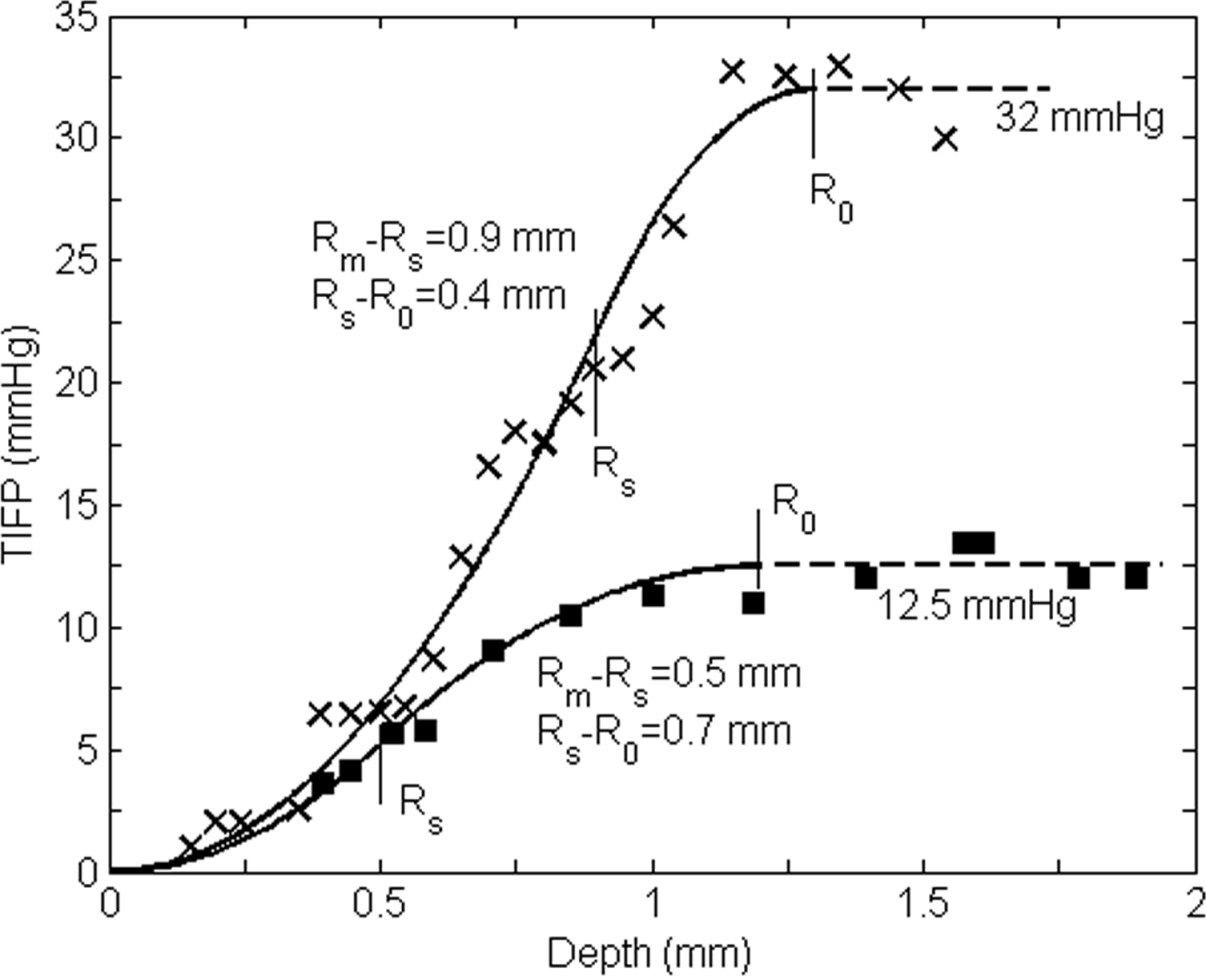
Comparison of our model for ‘embedded’ tumors with the experimental data from Figs 3A track 5 (lower) and 6 track 4 (upper, which represents approximately the average value of all readings from all tracks) in Ref. 1. The readings from these two tracks are distributed across all regions of the tumors.

Similarly, we can fit the experimental data from Fig 3A (track 5) of Ref. 1 well (for a mammary adenocarcinoma t.i. tumor). We choose these two sets of readings because they distribute completely from tumor central region to the periphery, and the readings are clear and can be digitalized accurately. Also, the readings from Fig 6 (track 4) in Ref. 1 almost represent the average effect of the data in this figure. The results are shown in Fig 7. The results of both Figs 6 and 7 show that our model can estimate the hydraulic conductivity *K*′ of a tumor or its IFP in a practical manner. Noninvasive tools such as contrast enhanced MRI can be applied to determine the size of the region from *R*_0_ to *R*_m_ (or *R*_s_) and the highest fluid velocity *u*(*R*_s_) as well as *d*_m_, and then used to estimate the hydraulic conductivity *K*′ of the tumor interstitium and TIFP.

Assuming that the conductivity within the periphery region is the same as that within the intermediary region, we find *K*′_upper_ = 8.45x10^-7^ cm^2^/mmHg, *K*′_lower_ = 7.80 x10^-7^ cm^2^/mmHg, and *u*_upper_(*R*_s_) = 4.16x10^-3^mm/s, *u*_lower_(*R*_s_) = 1.63x10^-3^ mm/s based on the data in Fig 7 by curve fitting. It shows that our model gives us information regarding the interstitial fluid flow and interstitium in the rim, e.g. the hydraulic conductivity *K*′, *d*_m_, *d*_0_ and *u*(*R*_s_). This provides a method for investigating tumor conditions at various stages.

## Discussion

The one-dimensional (1D) model may also be applied to approximate TIFP and fluid flow for a spherically symmetrical tumor when the ratio of the tumor rim to its radius *d*_m_/*R* is small. For example, theoretically, when *d*_m_/*R*_0_<5%, the relative error of the fluid flow (caused by the increase in surface area) is less than 10% when using the 1D approximation. Usually, the tumor periphery is around 2mm. A 1D model can give a good approximation for a $\bar{R}=10\text{mm}$ tumor and Δ*R*~±1mm.

There are some other noninvasive methods for measuring the velocity of interstitial fluid. For instance, Chary and Jain demonstrated the measurement of interstitial convection by fluorescence photobleaching [20]. Mostly, these measurements can only give the average fluid velocity at the tumor periphery. However, as having described above and in Ref. 9, for an isolated tumor, the fluid velocity at tumor surface is approximately twice of the average fluid velocity $u(R_{s})\approx 2\bar{u}$ since $\bar{u}\approx \frac{u(R_{0})+u(R_{s})}{2}=\frac{u(R_{s})}{2}$. In fact, when the interstitial fluid velocity of both isolated and embedded tumors is linearly distributed as

$u(r)=\{\begin{array}{cc} \frac{u(R_{s})(r-R_{0})}{R_{s}-R_{0}} & \left(R_{0}<r<R_{s}\right)\\ \frac{u(R_{s})(R_{m}-r)}{R_{m}-R_{s}} & \left(R_{s}<r<R_{m}\right) \end{array}$, the average velocity is $\bar{u}=\frac{\int_{R_{0}}^{R_{m}}u(r)dr}{\int_{R_{0}}^{R_{m}}dr}=\frac{u(R_{s})}{2}$. Therefore, the interstitial fluid velocity at the tumor surface may be estimated.

The hydraulic conductivity has a strong effect on TIFP, just as fluid velocity does. Aside from the methods for determining the hydraulic conductivity that we discussed above, we may also be able to determine the hydraulic conductivity indirectly by measuring other parameters such as the glycosaminoglycan (GAG) concentration, CGAG (g/100g tissue), and tissue water content z. It was found that the hydraulic conductivity correlates with GAG concentration as *K* = 4.6×10^−13^*C*_*GAG*_^−1.202^ [21, 22, 23]. The hydraulic conductivity in the normal host tissue is usually known. Thus, we may find the hydraulic conductivity in the tumor by comparing the GAG concentrations. Another method is based on a relationship between the hydraulic conductivity and the water content since *K* = *az*^*b*^, where *a* and *b* are constants that relate to interstitium conditions [21, 24]. Volumetric water content can be measured by using a near-infrared spectroscopy [25].

When there is no other drain (e.g. lymphatic system) and the fluid is enclosed, TIFP will be uniform and will reach an equilibrium state under this extreme condition. Thus, the fluid flow rate in such a region becomes zero (*J*_A_ = *J*_V_). We have (see Eq 17 in appendix)

$$p{\left(\text{TIFP}\right)}_{eq}=\frac{S_{A}L_{A}[p_{A}-\sigma_{A}(\pi_{A}-\pi_{Ai})]+S_{V}L_{V}[p_{V}-\sigma_{V}(\pi_{V}-\pi_{Vi})]}{S_{A}L_{A}+S_{V}L_{V}}.$$ (11)

When *S*_*A*_*L*_*A*_ = *S*_*V*_*L*_*V*_, the equilibrium value of TIFP is

$$p{\left(\text{TIFP}\right)}_{eq}=\frac{p_{A}+p_{V}-\sigma_{A}(\pi_{A}-\pi_{Ai})-\sigma_{V}(\pi_{V}-\pi_{Vi})}{2}.$$ (12)

Tumors have elevated interstitial fluid pressure due to high permeability of tumor blood vessels and poor drainage system. In fact, a poor drainage system may cause more serious problems. When the drainage system malfunctions, a small leakage source may cause high fluid pressure. If there are no venules or other drainage system such as lymphatics, the highest TIFP can reach is *p*_*m*_ = *p*_*A*_−*σ*_*A*_(*π*_*A*_−*π*_*Ai*_). For a tumor, the surface area of blood vessels in a unit volume *S*/V and their conductivity *L* are much larger than that in normal tissue [10]. Also, the oncotic pressure is elevated [26]. These are the factors that contribute to an increase in TIFP. As an example, here we cite the numerical values in Ref. 27: *p*_A_ = 35mmHg, *p*_V_ = 15mmHg, *π*_A_ = *π*_V_ = 28mmHg. For normal tissue: *π*_A*i*_ = 0.1 mmHg, *π*_V*i*_ = 3mmHg [27], *σ*_N_ = 0.91 [10]. Thus, *p*_m_≈9.6 mmHg and *p*(TIFP)_eq_ = 0.9 mmHg. For a tumor, *σ*_T_ = 0.82 [10], when the osmotic pressure of interstitial fluid π_A*i*_ and π_v*i*_, which should be approximately equal, are both elevated to approximately 20 mmHg [26], we get *p*_m_≈28.4 mmHg and *p*(TIFP)_eq_ = 18.4 mmHg.

High TIFP may collapse or create malfunctioning blood vessels in tumors. We assume that there are some blood vessels with vascular fluid pressure *p*_v1_, *p*_v2_, …*p*_vm_, where *p*_vm_ is the highest vascular pressure in the tumor. When the interstitial fluid pressure increases to a value that is higher than *p*_v1_, there is a positive net pressure difference Δ*p* = *p*-*p*_v1_>0 (Here we ignore the osmotic pressure) between the interstitial fluid and the blood plasma. In this case, blood vessel with vascular pressure *p*_v1_ cannot contribute to interstitial fluid (no fluid can come out from this blood vessel). Similarly, when *p*>*p*_v*i*_ (1<*i*<m), all blood vessels with *p*_v_<*p*_v*i*_ cannot contribute to interstitial fluid and will collapse or lose their function. The higher the TIFP, the more vessels will lose their function. This process will repeat until TIFP equilibrium *p*_eq_ has been reached. In this case, the amount of fluid leaked out equals the amount of interstitial fluid drained away. Depending on the condition of the tumor, *p*_eq_ can take on any value between 0 and *p*_vm_. If *p*_eq_ reaches *p*_vm_, no fluid can leak out from blood vessels within the tumor. This extreme condition gives maximal TIFP limit, which should be *p*_*Vm*_−*σ*_*V*_(*π*_*V*_−*π*_*Vm*_).

## Conclusion

The absolute value of TIFP can be approached by converting theoretical variables into measurable ones. We have presented a fairly realistic analysis of tumor exudate flow in tissue, with emphasis on the eventual application of a technique to noninvasively estimate TIFP. It is practical to select a specific direction for the approach to measuring TIFP, and it is viable for tumors of various shapes. However, velocity at the tumor surface as a measure of TIFP depends on other properties of the tumor, and particularly on its surround, with the parameters *R*_m_-*R*_0_ (or *R*_s_-*R*_0_) and tissue conductivity *K* of primary importance in defining the relation between TIFP and *u*(*R*_s_). These parameters can be measured. We provide a means of estimating tumor fluid velocity, and show that the ratio of fluid conductivity of a tumor to its rim size is independent of tumors. It shows that TIFP noninvasive measurement can be actualized. Also, this work provides a method for investigating tumor conditions at various stages by measuring the hydraulic conductivity and fluid flow within the tumor rim.

## Appendix

### Mathematical model derivation

Interstitial fluid flow in a tumor is formed by the sum of the net flow from various abnormally leaky blood vessels. Generally, it is expressed as [10,16]:

$$\nabla \cdot \vec{u}(\vec{r})=\frac{J_{s}}{V}-\frac{J_{L}}{V},$$ (13)

where *J*_s_/V (fluid source) is the volumetric net flow rate for fluid coming out of blood vessels, which is based on Starling’s law [28–30], and *J*_L_/V (fluid drain) the volumetric flow rate for fluid being reabsorbed into lymphatics.

Lymphatic vessels are present in most tissues and their function is to drain the interstitial fluid. Even though there are no lymphatics in the brain, there may be a similar system for draining the fluid [31]. The drainage term is usually described as [10]:

$$J_{L}=S_{L}L_{L}[p(\vec{r})-p_{L}(\vec{r})],$$ (14)

where *p* is the interstitial fluid pressure, *p*_L_ is the pressure in lymphatic vessels. We will use the subscripts ‘L’, ‘A’ and ‘V’ represent the corresponding counterparts of the lymphatic, arteriole and venule vessels. *L* represents the hydraulic conductivity of the vessels (m^2^s/kg), *S* the surface area of the vessels. Usually, arterioles and venules are distributed in pairs. Normally, the circulatory system processes 20 liters of blood on average per day through capillary filtration. Roughly 17 liters of the filtered plasma is reabsorbed into the blood vessels, and the remaining 3 liters in the interstitial fluid returns to the blood through the lymph system [32]. The fluid lost from, and gained by, plasma is balanced in normal tissue.

The microvascular sources of fluid follow Starling’s law [10,16] as follows.

$$J_{A}=S_{A}L_{A}(\vec{r})\left\{p_{A}(\vec{r})-p(\vec{r})-\sigma_{A}\left[\pi_{A}(\vec{r})-\pi_{Ai}(\vec{r})\right]\right\},$$ (15)

where *J*_A_ is the fluid flow rate for blood vessels. The flow rate of fluid that is reabsorbed by venules is expressed as:

$$J_{V}=S_{V}L_{V}(\vec{r})\left\{p(\vec{r})-p_{V}(\vec{r})+\sigma_{V}\left[\pi_{V}(\vec{r})-\pi_{Vi}(\vec{r})\right]\right\}.$$ (16)

To simplify the situation, we take the arteriole and the venule as a pair and state the net fluid flow rate:

$$J_{S}=J_{A}-J_{V}=S_{A}L_{A}[p_{A}-\sigma_{A}(\pi_{A}-\pi_{Ai})]+S_{V}L_{V}[p_{V}-\sigma_{V}(\pi_{V}-\pi_{Vi})]-(S_{A}L_{A}+S_{V}L_{V})p(\vec{r}).$$ (17)

where *p*_A_ (or *p*_v_) is the vascular plasma pressure, *σ*_A_ (*σ*_v_) the osmotic reflection coefficient, *π*_A_ (or *π*_v_) the osmotic pressure of the plasma, and *π*_*Ai*_ (*π*_v*i*_) the osmotic pressure of interstitial fluid [10], *S*/*V* the surface area of the vessels in a unit volume.

In many cases, a tumor boundary can be chosen so that it is locally one-dimensional. In this case, a 1D model can better describe the actual situation and easier for application. Combining Starling’s law with Darcy’s law (see appendix) for 1D case *u*(*r*) = -*K*d*p*(*r*)/d*r*, we have:

$$\{\begin{array}{cc} \frac{d^{2}p(r)}{dr^{2}}-\delta \cdot (p-p_{e})=0 & \left(R_{0}<r<R_{s}\right)\\ \frac{d^{2}p(r)}{dr^{2}}-\beta (p-p_{L})=0 & \left(R_{s}<r<R_{m}\right) \end{array}$$ (18)

where *K* is hydraulic conductivity of interstitium, $\beta =L_{L}\frac{S_{L}}{VK}$ (an index that reflects the strength of fluid source absorption), $\delta =\frac{L_{A}^{\prime }S_{A}+L_{V}^{\prime }S_{V}+L_{L}^{\prime }S_{L}}{VK^{\prime }}$, and $p_{e}=\frac{L_{A}^{\prime }S_{A}p_{A}+L_{V}^{\prime }S_{V}p_{V}+L_{L}^{\prime }S_{L}p_{L}-L_{A}^{\prime }S_{A}\sigma_{A}(\pi_{A}-\pi_{Ai})-L_{V}^{\prime }S_{V}\sigma_{V}(\pi_{V}-\pi_{Vi})}{L_{A}^{\prime }S_{A}+L_{V}^{\prime }S_{V}+L_{L}^{\prime }S_{L}}.$

The variables are primed (′) to signify that they relate to the fluid flow within the tumor.

The boundary and continuity conditions are: *p*(*R*_0_) = *p*_0_, *u*(*R*_0_) = -*K*′d*p*(*R*_0_)/d*r* = 0, *p*(*R*_s_^+^) = *p*(*R*_s_^-^) = *p*(*R*_s_), *K*d*p*(*R*_s_^+^)/d*r* = *K*′d*p*(*R*_s_^-^)/d*r* = -*u*(*R*_s_), *p*(*R*_m_) = *p*_∞_ (usually it is around 0 relative to the atmosphere since it is at the interface of normal tissue), *u*(*R*_m_) = −*K*d*p*(*R*_m_)/d*r* = 0.

Under the boundary and continuity conditions, Eq 18 can be solved. Now the theoretical variables have been converted into measureable variables. The solution of Eq 18 becomes

$$p(r)=\{\begin{array}{cc} p_{0}-\frac{2u(R_{s})\sinh^{2}[\sqrt{\delta }(r-R_{0})/2]}{K^{\prime }\sqrt{\delta }\sinh(\sqrt{\delta }d_{0})} & \left(R_{0}<r<R_{s}\right)\\ \frac{2u(R_{s})\sinh^{2}[\sqrt{\beta }(R_{m}-r)/2]}{\sqrt{\beta }K\sinh(\sqrt{\beta }d_{m})}+p_{\infty } & \left(R_{s}<r<R_{m}\right) \end{array}$$ (19)

where *d*_0_ = *R*_s_-*R*_0_ and *d*_m_ = *R*_m_-*R*_s_. Correspondingly, the tumor interstitial fluid velocity is:

$$u(r)=\{\begin{array}{cc} \frac{u(R_{s})\sinh[\sqrt{\delta }(r-R_{0})]}{\sinh(\sqrt{\delta }d_{0})} & \left(R_{0}<r<R_{s}\right)\\ \frac{u(R_{s})\sinh[\sqrt{\beta }(R_{m}-r)]}{\sinh(\sqrt{\beta }d_{m})} & \left(R_{s}<r<R_{m}\right) \end{array}$$ (20)

Here *u*(*R*_s_), *d*_0_ and *d*_m_ can be measured by administering a contrast agent that extravasates and then travels at the same velocity as the tumor exudate. Based on Eq 19, we obtain *p*(*R*_s_) and *p*_0_,

$$p(R_{s})=\frac{u(R_{s})}{\sqrt{\beta }K}\tanh\left(\frac{\sqrt{\beta }d_{m}}{2}\right)+p_{\infty },p_{0}=p(R_{s})+\frac{\tanh(\sqrt{\delta }d_{0}/2)u(R_{s})}{\sqrt{\delta }K^{\prime }}$$ (21)

When $\sqrt{\beta }d_{m}$ and $\sqrt{\delta }d_{0}$ are small, Eqs 19–21 reduce to Eqs 1–3.

With a loss of functioning lymphatics, the effective surface area of the drainage vessels becomes smaller and hence *β* becomes smaller. Thus, the value of *β* reflects tumor aggressiveness. The fluid flow (and the fluid velocity) at the tumor surface is determined by the fluid source and sink within the tumor. When there is no sink, even a slight vascular leakage may cause high TIFP *p*_0_, which can reach *p*_m_ (0<*p*_0_<*p*_m_) [9]. Many tumors, especially the malignant ones, may not have a functional drainage system within.
